# Assessing the effectiveness of social network interventions for adults with a diagnosis of mental health problems: a systematic review and narrative synthesis of impact

**DOI:** 10.1007/s00127-022-02242-w

**Published:** 2022-02-09

**Authors:** Helen Brooks, Angela Devereux-Fitzgerald, Laura Richmond, Penny Bee, Karina Lovell, Neil Caton, Mary Gemma Cherry, Bethan Mair Edwards, James Downs, Laura Bush, Ivaylo Vassilev, Bridget Young, Anne Rogers

**Affiliations:** 1 Mental Health Research Group, Jean McFarlane Building, Division of Nursing, Midwifery and Social Work, School of Health Sciences, Faculty of Biology, Medicine and Health, University of Manchester, Manchester Academic Health Science Centre, Manchester, M13 9PL UK; 2 Greater Manchester Mental Health NHS Foundation Trust, Manchester, UK; 3 Patient and Public Involvement Contributor, University of Manchester, Manchester, UK; 4 Department of Primary Care and Mental Health, Institute of Population Health, University of Liverpool, Liverpool, UK; 5 Linda McCartney Centre, Liverpool University Hospitals NHS Trust, Prescot St, Liverpool, UK; 6 Patient and Public Involvement Contributor, Cambridge, UK; 7 Faculty of Health Sciences, University of Southampton, Southampton, England; 8 Department of Public Health, Policy and Systems, Institute of Population Health, University of Liverpool, Liverpool, UK

**Keywords:** Mental health, Social networks, Systematic review, Narrative synthesis

## Abstract

**Background:**

Social connections have been linked to the genesis and amelioration of mental health problems and thus have potential therapeutic value.

**Purpose:**

To identify the current evidence base, assess risk of bias and synthesise findings on the effectiveness of social network interventions for people with mental health problems.

**Methods:**

Electronic databases (MEDLINE, Embase, PsycINFO, CINAHL, Cochrane Library, Web of Science, Scopus) and grey literature databases were systematically searched from inception to October 2021 using free text syntax combining synonyms for ‘mental health problems’ and ‘social network interventions’. Articles were eligible for inclusion if they reported data from randomised controlled trials on the effectiveness of interventions designed to improve social networks for adults (18+) with mental health problems. Papers were independently reviewed for inclusion with conflicts resolved through consensus. Included papers were quality assessed and data extracted and synthesized narratively. Risk of bias was assessed using the Cochrane Risk of Bias Tool.

**Results:**

Nine studies randomising 2226 participants were included. Four focused on those with a diagnosis of schizophrenia or psychosis, one on major depressive disorder and four included all types of mental health diagnoses. The current evidence base is of unclear quality. However, interventions which focused on supporting social activities appear to hold the most promise for enhancing social networks. Data on cost-effectiveness and research acceptability were limited, but suggest the potential economic feasibility of and acceptability for evaluating these interventions.

**Conclusion:**

There is emerging evidence that social network interventions can be effective in improving social connections for people with mental health problems. However, further evaluations with robust methodological approaches are required to inform evidence-based recommendations for health services.

**Supplementary Information:**

The online version contains supplementary material available at 10.1007/s00127-022-02242-w.

## Introduction

Mental health problems commonly occur with estimated lifetime prevalence rates of between 18 and 36% [1]. There are more disability-adjusted life years lost per year to mental health problems than any other health condition in the UK and costs to the individual, society and the economy are considerable [2]. Adults with severe mental health problems,^1^ such as schizophrenia and bipolar disorder, experience higher rates of multiple and more complex physical co-morbidities resulting in significantly reduced life expectancy of approximately 15–20 years [3, 4]. It is therefore imperative that health services are able to effectively and appropriately offer a range of support to people with mental health problems.

Social networks refer to the structure and function of a person’s social relationships and the nature of the ties that connect them [5]. A person’s social network constitutes the set of connections which have the capacity to link people to relationships and resources, and can aid, restrict and reshape the way in which mental health problems are managed [6]. These connections can take a variety of configurations covering the broad range of people, non-human agents, places, things and activities which may be involved in the everyday management of mental health problems [6, 7]. Increased connectivity is linked to the provision of social support, interpersonal contact and the mobilisation of resources [8] which acts to buffer stress through the provision of functional support as well as enhancing individual coping strategies [9]. However, this differs across groups and contexts [10, 11]. For example, high contact with social networks can increase levels of depressive symptoms for women if they are accompanied by a burden of obligation to provide large amounts of social support to others [9].

The Network Episode Model (NEM) provides a theoretical basis for understanding the contributions social networks make to the daily management of mental health problems [12, 13]. The NEM rejects individualistic approaches to mental health self-management and conceptualizes self-management instead as a collective activity that people do in conjunction with their social network [12, 13]. In line with other social network approaches, the NEM provides an analytic focus on the activation of social network ties in response to mental health problems and captures the dynamic social processes through which an individual manages their mental health problems with formal (mental health professionals) and informal (friends and family) networks [12, 14].

An individual’s ability to obtain support from their social networks and negotiate its acceptability to themselves and other members of their network is impacted by existing cultures and available network and individual resources [13, 15]. Social networks can provide a range of supports to an individual with a health condition, but such support is contingent on the availability of requisite knowledge, understanding and willingness to provide help within networks which is not always present or available to individuals [16]. Whilst cross-cultural social network studies are limited in number, research has demonstrated that network homogeneity and generalized trust within networks vary across cultures [17, 18]. Furthermore, research has demonstrated that propensity to seek help from others amongst older adults was dependent on informal logical and cultural rules which affected their decisions to help-seek, where to go to obtain support, whether it was available and adequate and interpretations of others willingness to provide help [19].

Diverse and supportive social networks have been found to have a positive influence on recovery for people with a diagnosis of severe mental illness [20]. However, people with mental health problems also tend to have smaller networks of poorer quality and configuration [21]. There is evidence too of variability in the availability of network resources over time, illness phases, illness severity and setting [22]. A mental health diagnosis has been shown to lead to an erosion of existing high-quality network connections in terms of size, diversity and access to resources [14]. However, network disruption can result in network reconfiguration with new network members replacing weak, lost or absent ties which may be more protective against psychological distress and of greater utility in managing a long-term condition [23]. The latter points to markers for the development and implementation of interventions aimed to improve mechanisms for mental health management and recovery.

Improving network-based strategies for managing everyday mental health and promoting social integration are necessary for accessing community-based support and promoting and engagement in meaningful activity [24]. In turn, social activity can lead to increased social network size and access to social capital^2^ [25] creating a virtuous circle [6]. Social networks can also mediate the effects of social isolation and loneliness, and enhance self-management [20, 26]. Thus, social network interventions which assist with eliciting preferences for connecting to meaningful, valued activities in domestic and local environments extends the availability of heterogenous support for the secondary prevention of mental health problems. [7, 27]. Whilst such interventions are successful for long-term physical health conditions (e.g., social prescribing), they have been slow to translate into mainstream mental healthcare despite the relevance of community engagement and integration for recovery [7].

This review aimed to provide a critical overview of the evidence base underpinning interventions designed to improve the quantity and quality of social networks of people with mental health problems. The acceptability, feasibility and cost-effectiveness of evaluating these social network interventions were explored by examining available data on evaluation adherence, attrition and cost evaluations within included trials.

### Review questions

What is the effectiveness of interventions designed to improve the quantity and quality of social networks of adults with mental health problems?

What are the factors that influence the effectiveness of social network interventions for people with mental health problems?

## Methods

The methods and reporting of this systematic review and narrative synthesis follow PRISMA (Preferred Reporting Items for Systematic Reviews and Meta-Analysis) guidance [28]. The protocol for the review is available from: https://www.crd.york.ac.uk/prospero/display_record.php?ID=CRD42020206490.

### Eligibility criteria

Only published research articles containing primary data were included in the review. Literature or systematic reviews on related topics were excluded, but reference lists examined for potentially relevant studies. Studies which recruited adult participants (aged 18+) with any form of self-report or professionally diagnosed mental health difficulty (excluding organic mental health difficulties such as dementia, learning disability and co-morbidities such as substance abuse) were considered, with no restrictions placed on the diagnosis, severity or length and stage of illness. In mixed samples, mean age requirement was a minimum of 18 years and 75% of identified samples required a primary diagnosis of mental health difficulties or self-reported emotional distress.

Eligible studies had to report on an intervention designed specifically to increase the quantity or quality of social networks. In the context of this review, social networks were defined as personal communities—the constellation of relevant relationships, activities and resources that are identified as important by an individual [29]. Eligible studies also had to include a measure of social network quantity or quality as either a primary or secondary outcome and utilise a randomised design with a comparison group. There were no restrictions placed on eligible studies based on language or date of publication. Non-English language articles were screened for eligibility by native speakers affiliated with the research team. See Table 1 for inclusion and exclusion criteria.

**Table 1:** Inclusion and exclusion criteria

Inclusion criteria	Exclusion criteria
Published journal articles, or dissertations	Duplicate
Primary data from studies which are designed directly to improve the quantity or quality of social networks (based on whole network approach)	Not primary data (e.g. opinion pieces, review articles, book chapters)
AND	
Include a measure of social network size and/or quality as primary or secondary outcome	
Adults with primary diagnosis of mental health problems or self-attribution/non-medical labelling (e.g. stress or emotional distress)	Only available in abstract format
In mixed samples, mean age must be 18 or over and 75% of sample must have primary diagnosis of mental illness (self-report or physician defined)	
Controlled trials (CT) and randomised controlled trials (RCT) including cluster-randomised trials	Single case studies
	Studies where primary diagnosis is substance misuse, autism, dementia, ADHD, cognitive impairment or spectrum disorders
	Patients without a primary diagnosis of mental health problems or self-attribution of mental difficulties (self-report or clinician diagnosis). In mixed samples 75% or more must have a primary diagnosis of mental illness or self-attribution of mental health difficulties
	Non-adult population: Mean age under 18
	Pharmacological interventions
	Intervention’s primary function is not related to improving the quantity and/or quality of social networks (conceptualized as a whole network approach). The following will be excluded:
	1. Dyadic interventions—couples, individual friendship interventions, family level only
	2. Individual level intervention—e.g. intervention which aims to improve individual social skills, social functioning/dysfunctioning, social cognitions, confidence in social interaction, perceptions about social interaction, social interaction intentions
	No measure of social network quantity or quality
	Qualitative studies, feasibility studies or uncontrolled or unrandomised trials
	Not accessible

### Search strategy

Seven electronic databases were searched (MEDLINE, Embase, PsycINFO, CINAHL, Cochrane Library, Web of Science, Scopus) were searched on the 29th of August 2020 from the earliest record and updated on the 5th October 2021. The search strategy was organised using the first two components of the PICO framework and was purposively broad to optimise retrieval (see “Appendix 1” for example search):

- ***Population***: People with a diagnosis of mental illness or self-reported emotional distress
- ***Intervention***: Social network

The search strategy was informed by published reviews, extant literature on social network interventions and following discussions with the wider authorship team. A draft version of the strategy was also subject to a PRESS review by an expert librarian [30].

To minimise the impact of publication bias, grey literature sites were searched including OpenGrey and EThoS. We contacted authors of identified conference abstracts for full manuscripts where these were not readily available through web search strategies. Reference lists of included manuscripts were also scrutinized for relevant studies. Additionally, we examined identified book chapters and literature reviews for relevant literature. Key journals were hand searched: Social Psychiatry and Psychiatric Epidemiology, BMC health services research, Journal of Mental Health, British Journal of Psychiatry and Lancet Psychiatry.

### Data selection and extraction

Search results were uploaded to the data management software Covidence (http://www.covidence.org) and duplicates removed. Titles and abstracts were double screened with conflicts resolved by a third reviewer. Eligibility assessments of full texts of potentially eligible manuscripts were undertaken by two reviewers with conflicts resolved by consensus. A systematic data extraction tool was developed using Excel into which quantitative data relating to the outcomes of interventions were extracted, along with data relating to study design, participants, adherence/attrition, cost-effectiveness and other relevant contextual factors. 30% of extractions and quality appraisals were checked for accuracy.

### Analysis

A meta-analysis of included studies including pooling the data and comparing mean differences of related outcomes (e.g., network size) was originally planned, but given the heterogeneity of included studies, this was not possible and a narrative synthesis was undertaken. This followed the stages outlined in the Guidance on the Conduct of Narrative Synthesis in Systematic Reviews [31].

An initial synthesis was undertaken by producing textual summaries of study characteristics (e.g., design, participants, intervention, and recruitment) in data extraction spreadsheets. Included studies were organised alphabetically in excel sheets, but allocated a colour code by type of intervention. We used ‘vote counting’ to describe the number of studies which demonstrated positive, negative or neutral results relating to social network outcomes [31]. The next stage of the narrative synthesis involved a consideration of the factors that influenced successful outcomes and any other included outcome measures. Prior to finalising the synthesis, all included studies were revisited along with the PRISMA checklist (“Appendix 2”) to ensure that relevant data were not omitted from the presentation of results.

## Results

**Fig. 1:**
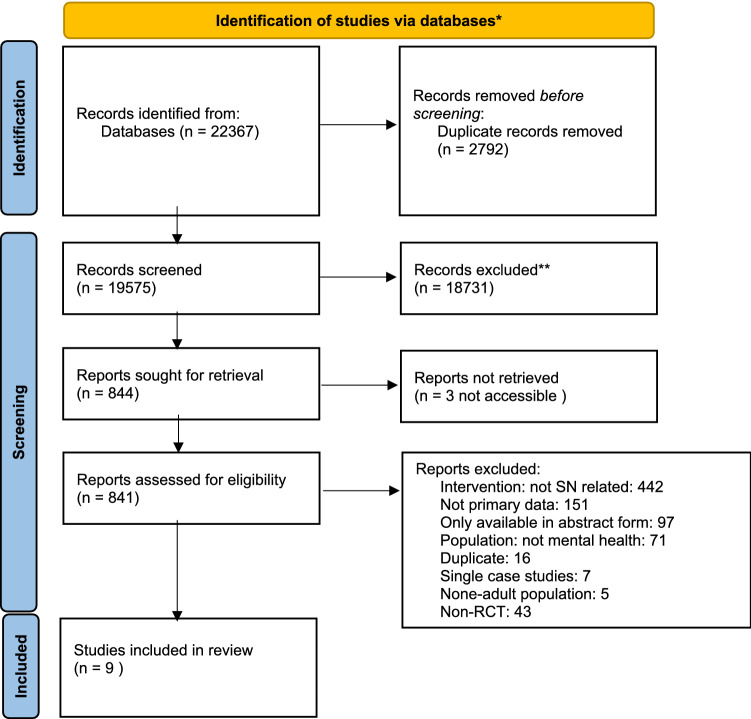
PRISMA 2020 flow diagram

The results of the search, screening and selection for final included studies can be found in Fig. 1. Searches generated 22,367 hits of which 2792 duplicates were removed. The majority of the remaining 19,575 were excluded at title and abstract screening. Of the 841 full texts screened for eligibility, 9 were included in the systematic review. The main reasons for exclusion were interventions not being designed with an explicit focus to improve social networks, non-mental health populations and non-RCT designs (Fig. 1).

### Description of included studies

The studies reported were heterogenous in terms of intervention format and delivery, outcome measures and length of follow-up. Descriptions of included studies can be found in Supplementary File 1.

#### Study characteristics

Three studies were carried out in the USA [32–34], two in the UK [35, 36] and one each in Denmark [37], Italy [38], Ireland [39], and the Netherlands [40]. All studies reported on the results of interventions for formal mental health diagnoses and no studies included those with self-reported emotional distress. Four studies included only those with a diagnosis of schizophrenia or psychosis [35, 37, 38, 40] with one recruiting only those with first episode psychosis [37]. One study exclusively comprised people with major depressive disorder [33], and the remaining studies included people with broader diagnostic categories of mental illness described as enduring mental health problems [39], AXIS I and II disorders (using DSM-III-R), [34], AXIS I Psychotic or mood disorders (DSM version not reported) [32] or included all forms of mental health conditions [36]. Most studies utilised broad conceptualisations of social networks incorporating both quantity and quality of social network support [32–36, 38–40]. Only one used social network size as the sole proxy for social network contributions with the authors acknowledging this as a limitation [37].

#### Participant characteristics

Included studies randomised a total of 2,226 participants across intervention and control conditions. The average age of included participants was 35.7 years. On average, 49.4% of participants were female. Only 5 reported ethnicity data with White participants accounting for 47% of participants across these included studies. Black participants accounted for 34.4%, Hispanic participants for 6.2%, Asian participants for 1% and other ethnicity groups accounting for 11.4%.

#### Intervention characteristics

Included studies recruited from formal health services (community and inpatient settings) and all interventions were delivered in the community. Five were delivered/facilitated by health professionals [33–35, 37, 40], three by lay volunteers including peers or family members [32, 36, 38] and one by a combination of professional and lay facilitators [38]. Allocated control conditions were mostly treatment as usual [32–35, 37, 38] or wait list control [40]. Active comparators included financial stipend [39] and personal recovery workbook [36].

Intervention duration ranged from 3 to 12 months with follow-up data collection periods ranging from 3 to 24 months. All interventions were delivered face-to-face. Interventions mostly comprised supported social activity/community; one explicitly aimed to develop a friendship between participant and facilitator [39]; and one included financially supported socialisation [39]. One intervention was a closed peer support group with a primary aim of improving participants’ social networks [40]. Two interventions involved one-to-one work with participants using either cognitive behavioural therapy [33] or recovery-focused activities aiming to enhance social networks [36]. Three interventions were assertive community treatment interventions with a social network focus which included family members and friends in the treatment process [34, 35, 37].

### Risk of bias

Details of the risk-of-bias assessments drawing on the Cochrane Risk of Bias Tool [41] are presented in Supplementary File 1 which incorporated six domains where bias could be introduced into trial design. No studies were assessed as being “low risk of bias”. Five studies were assessed as being high risk and the other four did not provide sufficient information for risk-of-bias assessments to be undertaken. Therefore, the proportion of information from studies at high risk of bias is considered sufficient to affect the interpretation of results [41].

### Clinical effectiveness

Summary information on clinical effectiveness, effect size and study quality can be found in Tables 2, 3, 4. Interventions were categorised into four types based on core activities: supported social activity, peer support, assertive community treatment and one-to-one interventions.

**Table 2:** Overview of study quality, clinical significance and effect sizes for social network measures

Study ref	Risk of bias	Intervention descriptor (*n*)	Comparator descriptor (*N*)	Outcome measure	Differences between groups—effect direction + ,−, 0	Standardised effect size (or for dichotomous variables and effect size for continuous variables). longest follow-up
Terzian, 2013	High	Supported social activity (*n* = 173)	Standard care (*n* = 172)	A social network improvement—defined as an increase in number, frequency, importance, or closeness of relationships	+	OR: 1.8. 95% CI: 1.2–2.9
Sheridan, 2015	High	Supported social activity, volunteer partner, stipend (*n* = 32)	Stipend only (*n* = 38)	Practitioner Assessment of Network Type	0	N/A
				Social and Emotional Loneliness Scale for Adults		
Rivera, 2007	Unclear	Peer supported social activity (*n* = 70)	Standard case management (*n* = 66)	Pattison Network Inventory: Total number of social contacts	+	Compared to usual clinical care: Medium-effect size: 0.470497
			Usual clinical care (*n* = 67)	Social network size	0	
				Density	0	
				Reciprocity	0	
Castelein, 2008	High	Closed peer support group (n = 56)	Waiting list control (*n* = 50)	Personal Network Questionnaire (PNQ)	+	Participants had a significant increase in contact with peers outside of the sessions. Not possible to calculate effect size
				The Social Support List (SSL)	+	Participants had a significant increase in esteem support (i.e. asked for advice, received a compliment, asked for help) Small effect size: 0.390877
Thorup, 2006	High	Assertive community treatment (*n* = 194)	Standard care (n = 153)	Social network size	0	N/A
Tempier et al. 2012	Unclear	Assertive community treatment (*n* = 57)	Standard care (*n* = 50)	Social network size	+	Medium-effect size: 0.609451
				Functional adequacy of social networks	0	N/A
Calsyn, 1998	Unclear	Assertive community treatment and community workers	Assertive community treatment	Network size: Size of professional network	+	No sample size provided
		(sample sizes not provided for each condition)	Brokered condition (standard case management)	Size of natural network	0	N/A
				Receipt of material assistance	+	No sample size provided
				Emotional, advice, recreational and conflict dimensions	0	N/A
				Qualitative measures of social relationships Interviewer rated network support:	0	N/A
				Professional network	+	No sample size provided
				Natural network	0	N/A
Johnson et al., 2018	High	One-to-one recovery focussed intervention (*n* = 220)	Recovery workshop (*n* = 219)	Social network size	0	N/A
				Los Angeles (UCLA) Loneliness Scale	0	N/A
Ammerman, 2013	Unclear	One-to-one cognitive behavioural therapy (*n* = 47)	Standard home visiting (*n* = 46)	Social Network Index—3 sub-scales:	0	N/A
				Social network size	0	N/A
				Network diversity	0	N/A
				Embeddedness	+	Medium-effect size: 0.65
				Interpersonal Support Evaluation List	+

**Table 3:** Overview of study quality, clinical significance and effect sizes for mental health outcomes

Study refs	Risk of bias	Intervention descriptor (*n*)	Comparator descriptor (*N*)	Outcome measure	Differences between groups—effect direction + ,−,0	Standardised effect size (or for dichotomous variables and effect size for continuous variables). longest follow-up
Terzian et al.,	High	Supported social activity (*n* = 173)	Standard care (*n* = 172)	Brief Psychiatric Rating Scale and Global Assessment of Functioning scores (a reduction of more than 3 points in the BPRS score or an increase of more than 5 in the GAF score were classified as clinical improvement)	0	N/A
Sheridan, 2015	High	Supported social activity, volunteer partner, stipend (*n* = 32)	Stipend only (*n* = 38)	Beck’s Depression Inventory	0	N/A
Rivera, 2007	Unclear	Peer supported social activity (*n* = 70)	Standard case management (*n* = 66) Usual clinical care (*n* = 67)	Service use Brief Symptom Inventory	0	N/A
Castelein, 2008	High	Closed peer support group (*n* = 56)	Waiting list control (*n* = 50)	None included	N/A	N/A
Thorup, 2006	High	Assertive community treatment (*n* = 194)	Standard care (*n* = 153)	None included	N/A	N/A
Tempier et al. 2012	Unclear	Assertive community treatment (*n* = 57)	Standard care (*n* = 50)	Positive and Negative Syndrome Scale (PANSS)	+	Medium-effect size: 0.548072
				Social functioning was assessed by using the Global Assessment of Functioning (GAF)	+	Medium-effect size: 0.567348
Johnson et al., 2018	High	One-to-one recovery focussed intervention (*n* = 220)	Recovery workshop (*n* = 219)	Readmission to an acute service	+	OR: 0·66 95% CI 0·43–0·99
				Days in acute care	0	
				Questionnaire on the Process of Recovery Illness	0	
				Management and Recovery Scale	0	
				Brief Psychiatric Rating Scale	0	
Ammerman, 2013	Unclear	One-to-one cognitive behavioural therapy (*n* = 47)	Standard home visiting (*n* = 46)	Brief Symptom Inventory	+	Medium-effect size: 0.73

**Table 4:** Overview of study quality, clinical significance and effect sizes for other outcomes

Study refs	Risk of bias	Intervention descriptor (*n*)	Comparator descriptor (*N*)	Outcome measure	Differences between groups—effect direction + ,−,0	Standardised effect size (or for dichotomous variables and effect size for continuous variables). longest follow-up
Terzian et al.,	High	Supported social activity (*n* = 173)	Standard care (*n* = 172)	Self-care	0	N/A
				Activities of daily living	0	N/A
				Hospitalisations	0	N/A
Sheridan, 2015	High	Supported social activity, volunteer partner, stipend (*n* = 32)	Stipend only (*n* = 38)	Rosenberg’s Self-Esteem Scale	0	N/A
Rivera, 2007	Unclear	Peer supported social activity (*n* = 70)	Standard case management (*n* = 66)	Behavioural Health Care Rating of Satisfaction	0	N/A
			Usual clinical care (*n* = 67)	Lehman Quality of Life Inventory	0	N/A
Castelein, 2008	HIGH	Closed peer support group (*n* = 56)	Waiting list control (*n* = 50)	Mental Health Confidence Scale (MHCS)	0	N/A
				Rosenberg’s Self-Esteem Scale	0	N/A
				WHO Quality of Life (WHO QoL) Bref	0	N/A
Thorup, 2006	High	Assertive community treatment (*n* = 194)	Standard care (*n* = 153)	None reported		
Tempier et al. 2012	Unclear	Assertive community treatment (*n* = 57)	Standard care (*n* = 50)	None reported		
Johnson et al., 2018	High	One-to-one recovery focussed intervention (*n* = 220)	Recovery workshop (*n* = 219)	Client Satisfaction Questionnaire	0	N/A
Ammerman, 2013	Unclear	One-to-one cognitive behavioural therapy (*n* = 47)	Standard home visiting (*n* = 46)	Not reported		

#### Social network quality and quantity

##### Structured support for undertaking social activity

All three interventions in this category provided some evidence of the potential impact of structured support for socialising in terms of improving the quantity and quality of social networks [32, 38, 39]. The two interventions which had a usual care comparator demonstrated significant improvements in social networks at 12-month (medium-effect size: 0.47) [32] and 24-month follow-up (OR: 1.8)—[38]) in the intervention groups. The third which compared supported socialisation with a financial stipend to the provision of finical stipend only demonstrated significant improvement in both groups which favoured the intervention, but did not reach significance. All three interventions targeted severe and enduring mental health problems such as psychosis and schizophrenia.

Terzian and colleagues targeted people under 45 years. Those with poor social networks (defined as five relationships) demonstrated a significant social network improvement (defined as an increase in number, frequency, importance, or closeness of relationships) at both 1-year (OR 1.8, 95% CI 1.2–2.8) and 2-year follow-up (OR 1.8, 95% CI 1.2–2.9) for the supported socialisation intervention which was delivered by professionals and lay facilitators (friends/family) [38]. The intervention was most effective for people who also demonstrated improvement in clinical, work or daily activity outcomes. For those who had no such improvement in these outcomes, the authors reported no impact of the intervention on social networks. The study reported that participants attached greater value to more distal ties than close friendships or confiding relationships [38].

Sheridan et al. [39] compared the effectiveness of a monthly stipend to support weekly leisure/social activity vs. monthly stipend plus supported social activity and friendship activities facilitated by people with no connection to mental health services. There were no significant differences between groups on social network outcome measures. However, there was a reduction in the number of people who had the most vulnerable types of networks post-intervention and increases in the weekly number of social contacts with friends in both groups [39]. Over the 10-month follow-up period, both groups demonstrated significant increases in social activities (e.g., going to the cinema, enjoying a conversation which favored the partnered group, but did not reach statistical significance), and increased social functioning, and decreased social loneliness [39].

Finally, Rivera et al. (2007) examined the outcomes of consumer-assisted case management, non-consumer-assisted case management and standard clinic-based care. Consumer-assisted case management involved matching service users with peers on socio-demographics and mental health experience to provide supported socialisation. The study found a significant increase (medium-effect size: 0.47) in the number of contacts from baseline to 12-month follow-up in consumer-assisted case management [32]. This effect was suggested to be due to increased contact with peer volunteers and professional staff, rather than with family/friends outside of health services. However, there were significant improvements in all conditions for other network variables including network density, numbers of people who helped the participant, and number of people who were helped by the participant.

##### Peer support

Castelein et al. [40] evaluated the effectiveness of a closed peer support group. This study demonstrated a significant improvement (small effect size: 0.4) in terms of contacts with peer facilitators outside of intervention activities and on ‘esteem support’ (e.g., asking for help, support and advice, receiving complements). However, esteem support did not extend to the number of other kin/non-kin relationships or to other measures of network quality or satisfaction with network support [40]. People who experienced greater distress from positive symptoms and a longer duration of illness were more likely to report improved social networks at follow-up, in contrast to those with higher distress from negative symptoms who were significantly less likely to improve their social networks [40].

##### Assertive community treatment

The three assertive community treatment interventions (Calsyn et al. [34], Tempier et al. [35], Thorup et al. [37]) demonstrated impact in terms of increasing the number of professionals in networks [34] and the number of significant others at 18-month follow-up (medium-effect size: 0.6) [35]. Increases in the size of lay/informal networks were identified as a trend in other studies, but did not reach statistical significance [34]. Other studies reported no differences between control and intervention groups at follow-up in relation to social network quantity, quality or the amount of social support received [34, 37]. Increased social network size at follow-up was closely related to younger age, being female, having completed A-levels, less negative symptoms, larger network size at entry [37].

##### One-to-one interventions

The two one-to-one interventions demonstrated no significant impact on social networks [33, 36], though one reported medium (0.7) effect sizes for increases in social support for those in the intervention group suggesting some improvement to social network quality outcomes [33].

#### Other outcome measures

Interventions demonstrating impact in terms of improving the quantity or quality of social networks either did not report other health-related outcome measures [34] or did not demonstrate significant intervention superiority [32, 38, 39]. However, both groups (stipend and stipend plus peer supported socialisation) in the trial by Sheridan et al. reported a significant reduction in depression symptomatology over the 10-month follow-up period (*p* = 0.001) [39].

Other included interventions demonstrated significant impact in terms of symptomatology [33, 35, 40], psychological distress [33], self-esteem [33], functioning [35], readmission to mental health services [36] and satisfaction with care [36]. Medium-reported effect sizes ranged from 0.5 to 0.7 demonstrating the direct impact of interventions aiming to improve network engagement may be independent from observable changes in social networks.

### Economic evaluation

Only two studies reported data pertaining to the evaluation of the costs associated with the interventions [38, 40] with only one of these constituting a formal cost assessment [40]. Castelein et al. [40] registered all prospective healthcare costs for included participants and other costs associated with the intervention. Their mixed model analysis demonstrated no significant differences in the mean total costs for both the intervention and control group. Terzian included an economic assessment and concluded their intervention had the potential to be readily included in routine care without the need for supplementary resources [38].

### Research feasibility and acceptability of evaluating social network interventions

Of the 2,226 participants randomised, 586 (26%) dropped out of the research follow-up and 1640 completed data collection at all time points. The lowest drop-out rates were identified in the supported socialisation intervention delivered by health professionals and natural facilitators [38] and the closed peer support intervention [40]. The highest withdrawal rates were found in the one-to-one recovery-focused intervention [36] and the supported socialisation with friendship intervention [40]. For the one-to-one recovery-focused intervention, the 18-month follow-up response rate was considered a limitation, but reasons for withdrawal were not discussed [36]. For the supported socialisation intervention, reasons for the high level of withdrawal which were concentrated in the intervention group included the emotional and practical demands of establishing and sustaining new friendships initiated during the intervention [39].

Most studies reported that participants and facilitators viewed the intervention positively with adherence not explained by demographic or clinical characteristics [34, 36, 40]. Data from associated process evaluations were lacking.

### Patient and public involvement

No included studies provided detail on any formal patient and public involvement in either the design and delivery of the intervention or the randomised controlled trial. One study reported that an intervention was adapted following feedback from participants [33].

## Discussion

We undertook a narrative synthesis of empirical data from randomised controlled trials to systematically examine whether social network interventions are effective in enhancing the quantity and quality of social networks for people with mental health problems. Despite the small number and inadequacies of the included studies, our analysis points to most promise of interventions which provide support for social activities supporting the findings of previous research [42, 43]. However, most studies (7/9) lacked requisite information to undertake the assessments of potential bias on at least one quality domain. Information on adherence to the candidate interventions was lacking in 7/9 studies, and detail on blinding of outcome assessors was omitted in 4/9 studies or assessed as high risk in another. Future research would benefit from more detailed descriptions of methods in order for quality assessments to be fully undertaken and to allow definitive conclusions about optimal treatments to be derived.

For interventions which were effective in enhancing social networks, effect sizes were generally small to moderate when compared to usual care. These benefits did not routinely translate to improvements in mental health outcomes, suggesting that more research is needed to investigate whether there is an embedding period beyond the follow-up periods in included studies [32, 38, 39]. Other studies which were not effective in improving social networks did provide evidence of demonstrable impact in a range of other outcomes (in particular assertive community outreach and one-to-one treatment) suggesting a more direct mode of action but one that might not be sustainable post-treatment without associated network improvements [33, 35, 36, 40]. More research is required to provide an in-depth understanding of the mechanisms underpinning such impacts [44]. For example, the extent to which specific properties of networks such as homophily (being together with similar others), weak tie contact or the opportunity for reciprocity might be candidate elements to include in future network interventions. One option is to undertake mixed-method systematic reviews to synthesise qualitative data which could be explored in relation to the available quantitative data on outcomes to identify potential mechanisms or determinants of behaviour change. This would allow hypotheses to be generated for future testing and would inform logic models for social network interventions to allow for theorizing to be initiated in terms of what works best for whom in what circumstances [45, 46]. Existing measures of social network size and quality may also not reflect more subtle changes in network enhancement (availability of acceptable support or collective efficacy within networks) which indicates the need for more sensitive measures of social networks. The development of a Patient-Reported Outcome Measure might allow for the quantification of social network structural and functional aspects by incorporating the perspectives of service user and carers themselves [47].

Only a small number of included studies highlighted factors associated with the effectiveness of social network interventions. However, there was emerging evidence of the potential influence of a number of factors. For example, people with better clinical prognoses experienced greater improvements to their social networks [38] as did people with better quality networks at baseline [37]. Older age and being male were negatively associated with enhanced social networks at follow-up periods [37]. This may reflect the findings in the wider literature which indicates that older people and men tend to have smaller social networks of poorer quality more generally and are more likely to face more challenges developing and sustaining social networks over time [48, 49]. Negative symptoms were associated with poorer quality of networks at follow-up [37, 40], whereas distress from positive symptoms was associated with enhanced social networks at follow-up [37]. Future research is required to examine mediating factors to guide future implementation [46].

Most interventions limited the types of network members included within networks to friends and family members and failed to incorporate alternative forms of network members identified as important to mental health management in the wider literature, including weak ties [7, 50], valued places, objects and activities [6, 7] and companion animals [51, 52]. This broader view of social network support was supported by the value attached to distal relationships by participants. Furthermore, complexities associated with establishing and maintaining friendships leading to withdrawal, and the equivalence in social networks of those involving financial stipend ± peer support [39], lend further support to the value of alternative network members [7].

Despite a number of included studies, reporting that the research processes were well received by participants and facilitators which suggest a willingness to participate in such evaluations [34, 36, 40], in-depth data on the feasibility of evaluating social network interventions were not reported and studies had an average drop-out rate in excess of 26%. There were also limited data in included manuscripts about intervention acceptability. The Medical Research Council’s guidance for the evaluation of complex intervention recommends the undertaking of process evaluation to understand the mechanisms through which interventions work and future evaluation should incorporate these in the design of evaluative studies [45]. Future research should also consider the minimum intervention period required, potential for intervention latitude—the freedom to undertake local adaptation which is critical for maximising intervention effect, ownerships and for promoting sustainability [53]—and consider the reasons for participant withdrawal and how to mitigate against these to inform intervention development and implementation.

Peer support in the design and delivery of mental health services has been shown to reduce hospital admissions and drive recovery-focused care, a core value enshrined in global health policy [54]. However, evidence in terms of using peer-supported socialisation outside formal mental health services, however, is mixed [55]. This review contributes to this debate by demonstrating that professional facilitators appear best placed to bring about increases in professional support within networks and peer workers are effective in developing relationships with service users that endure outside of health services [32, 40]. The review also supports other studies which have shown that, to make changes to whole networks and improve socialisation in the wider community, efforts are best focused outside of mental health services. This includes interventions drawing on lay workers that have no connection to formal service provision [56]. Potential reasons for this evident in the wider literature include expectations of acceptance by peers with similar experiences which were not realised in practice, limited instrumental resources and social networks of peer facilitators and the community stigma associated with mental health problems [56] Future research is required to understand optimal facilitation and what characteristics, training and support plans are required to effectively facilitate social network interventions for people with mental health problems [57].

This systematic review draws strength from the rigorous search strategy and extraction methods. To mitigate against bias, researchers independently screened all potentially eligible manuscripts with any conflicts resolved through consensus. Our research team included a range of health services researchers, practitioners and five patient and involvement (PPI) contributors. This enhanced the quality of the review in terms of the development of search terms and classification of interventions and resultant interpretation and presentation of findings. Specifically, PPI contributors suggested extracting information relating the PPI in included studies which illuminated the dearth of such activities, provided additional search terms not originally considered, enabled the context of interventions to be understood in more depth to support classification, and supported the development of recommendations for future research and practice. Analysis was hindered by the clinical and methodological heterogeneity of included studies and a lack of shared definitions and theoretical underpinnings of the term ‘social network’ and related concepts within manuscripts. The majority of included studies focused on schizophrenia or other forms of psychosis and generalisability to other mental health problems is unclear. There were a lack of economic data in included studies which meant that a full analysis in this regard was not possible. Despite employing no country or language restrictions, all identified studies were limited to USA and Europe which is an important limitation given that social networks are embedded in and reflect local cultures and contexts. Further research is required which incorporates wider geographical and cultural diversity.

## Conclusion

We found preliminary evidence that social network interventions can be effective in improving social networks for people with mental health problems. However, this review demonstrates that evidence for social network interventions for people with mental health problems is in its infancy and further rigorous evaluation is required to inform evidence-based recommendations for health services. Future research should incorporate nested process evaluations to understand and optimise implementation, adequate patient and public involvement to increase intervention uptake and acceptability and high-quality cost data to allow in-depth economic modelling to be undertaken.

### Electronic supplementary material

Below is the link to the electronic supplementary material.

Supplementary file1 (DOCX 35 kb)

## Appendix 2: PRISMA Checklist

**Table:**

Section and Topic	Item #	Checklist item	Location where item is reported
**TITLE**			
Title	1	Identify the report as a systematic review	Page 1
**ABSTRACT**			
Abstract	2	See the PRISMA 2020 for Abstracts checklist	Page 2
**INTRODUCTION**			
Rationale	3	Describe the rationale for the review in the context of existing knowledge	Pages 3–4
Objectives	4	Provide an explicit statement of the objective(s) or question(s) the review addresses	Pages 4–5
**METHODS**			
Eligibility criteria	5	Specify the inclusion and exclusion criteria for the review and how studies were grouped for the syntheses	Table 1
Information sources	6	Specify all databases, registers, websites, organisations, reference lists and other sources searched or consulted to identify studies. Specify the date when each source was last searched or consulted	Page 6
Search strategy	7	Present the full search strategies for all databases, registers and websites, including any filters and limits used	Appendix 1
Selection process	8	Specify the methods used to decide whether a study met the inclusion criteria of the review, including how many reviewers screened each record and each report retrieved, whether they worked independently, and if applicable, details of automation tools used in the process	Pages 6–7
Data collection process	9	Specify the methods used to collect data from reports, including how many reviewers collected data from each report, whether they worked independently, any processes for obtaining or confirming data from study investigators, and if applicable, details of automation tools used in the process	Pages 6–7
Data items	10a	List and define all outcomes for which data were sought. Specify whether all results that were compatible with each outcome domain in each study were sought (e.g. for all measures, time points, analyses), and if not, the methods used to decide which results to collect	Pages 6–7 and Supplementary File 1
	10b	List and define all other variables for which data were sought (e.g. participant and intervention characteristics, funding sources). Describe any assumptions made about any missing or unclear information	Pages 6–7 and Supplementary File 1
Study risk-of-bias assessment	11	Specify the methods used to assess risk of bias in the included studies, including details of the tool(s) used, how many reviewers assessed each study and whether they worked independently, and if applicable, details of automation tools used in the process	Page 8 and Supplementary File 1
Effect measures	12	Specify for each outcome the effect measure(s) (e.g. risk ratio, mean difference) used in the synthesis or presentation of results	Page 8 and Table 2
Synthesis methods	13a	Describe the processes used to decide which studies were eligible for each synthesis (e.g. tabulating the study intervention characteristics and comparing against the planned groups for each synthesis (item #5))	Page 7
	13b	Describe any methods required to prepare the data for presentation or synthesis, such as handling of missing summary statistics, or data conversions	N/A
	13c	Describe any methods used to tabulate or visually display results of individual studies and syntheses	Page 7
	13d	Describe any methods used to synthesize results and provide a rationale for the choice(s). If meta-analysis was performed, describe the model(s), method(s) to identify the presence and extent of statistical heterogeneity, and software package(s) used	Page 7
	13e	Describe any methods used to explore possible causes of heterogeneity among study results (e.g. subgroup analysis, meta-regression)	N/A
	13f	Describe any sensitivity analyses conducted to assess robustness of the synthesized results	N/A
Reporting bias assessment	14	Describe any methods used to assess risk of bias due to missing results in a synthesis (arising from reporting biases)	Page 8
Certainty assessment	15	Describe any methods used to assess certainty (or confidence) in the body of evidence for an outcome	N/A
**RESULTS**			
Study selection	16a	Describe the results of the search and selection process, from the number of records identified in the search to the number of studies included in the review, ideally using a flow diagram	Figure 1
	16b	Cite studies that might appear to meet the inclusion criteria, but which were excluded, and explain why they were excluded	Pages 5–6 and Table 1
Study characteristics	17	Cite each included study and present its characteristics	Supplementary File 1
Risk of bias in studies	18	Present assessments of risk of bias for each included study	Supplementary File 1
Results of individual studies	19	For all outcomes, present, for each study: (a) summary statistics for each group (where appropriate) and (b) an effect estimate and its precision (e.g. confidence/credible interval), ideally using structured tables or plots	Supplementary File 1 and Table 2
Results of syntheses	20a	For each synthesis, briefly summarise the characteristics and risk of bias among contributing studies	Page 8
	20b	Present results of all statistical syntheses conducted. If meta-analysis was done, present for each the summary estimate and its precision (e.g. confidence/credible interval) and measures of statistical heterogeneity. If comparing groups, describe the direction of the effect	N/A
	20c	Present results of all investigations of possible causes of heterogeneity among study results	N/A
	20d	Present results of all sensitivity analyses conducted to assess the robustness of the synthesized results	N/A
Reporting biases	21	Present assessments of risk of bias due to missing results (arising from reporting biases) for each synthesis assessed	N/A
Certainty of evidence	22	Present assessments of certainty (or confidence) in the body of evidence for each outcome assessed	N/A
**DISCUSSION**			
Discussion	23a	Provide a general interpretation of the results in the context of other evidence	Pages 14–16
	23b	Discuss any limitations of the evidence included in the review	Page 16
	23c	Discuss any limitations of the review processes used	Page 16
	23d	Discuss implications of the results for practice, policy, and future research	Pages 14–16
**OTHER INFORMATION**			
Registration and protocol	24a	Provide registration information for the review, including register name and registration number, or state that the review was not registered	Page 5
	24b	Indicate where the review protocol can be accessed, or state that a protocol was not prepared	Page 5
	24c	Describe and explain any amendments to information provided at registration or in the protocol	Page 5
Support	25	Describe sources of financial or non-financial support for the review, and the role of the funders or sponsors in the review	Page 17
Competing interests	26	Declare any competing interests of review authors	Page 17
Availability of data, code and other materials	27	Report which of the following are publicly available and where they can be found: template data collection forms; data extracted from included studies; data used for all analyses; analytic code; any other materials used in the review	Page 17

*From:* Page MJ, McKenzie JE, Bossuyt PM, Boutron I, Hoffmann TC, Mulrow CD, et al. The PRISMA 2020 statement: an updated guideline for reporting systematic reviews. BMJ 2021;372:n71. https://doi.org/10.1136/bmj.n71

For more information ((see Fig. 1)

## Footnotes

1 Defined as mental health problems which substantially interfere with or limits functional or occupational activities 3.Public Health England, *Severe mental illness (SMI) and physical health inequalities: briefing*. 2018, Public Health England: London.

2 Defined as “Features of social organisation, such as trust, norms and networks that can improve the efficiency of society by facilitating coordinated actions” 25. Putnam RD., R. Leonardi, and R. Nanenetti, *Making democracy work: civic traditions in modern Italy.* 1993, Princeton: Princeton University Press.

## Declarations

### Availability of data and materials

Data sharing is not applicable to this article as no datasets were generated or analysed during the current study. All extracted data are available with the manuscript.
