# Supplementary material for: Assessing the effectiveness of social network interventions for adults with a diagnosis of mental health problems: a systematic review and narrative synthesis of impact
Source: Soc Psychiatry Psychiatr Epidemiol. 2022 Feb 9;57(5):907–25. doi: 10.1007/s00127-022-02242-w (PMC9042995; doi:10.1007/s00127-022-02242-w)
Supplement: Supplementary file 1 — Supplementary file1 (DOCX 35 kb) [file 127_2022_2242_MOESM1_ESM.docx]

| **Context** | | | | | | | | | |
| --- | --- | --- | --- | --- | --- | --- | --- | --- | --- |
| **STUDY ID** (Author last name, year) | **COUNTRY** | **RECRUITMENT METHOD** | **RECRUITMENT SETTING** | **DELIVERY SETTING** | **DATA COLLECTION** | **FUNDING** | **INTERVENTION TYPE AND CONTROL** | **INTERVENTION LENGTH** | **LONGEST FOLLOW-UP PERIOD** |
| Terzian et al 2013 | Italy | Health professional at community mental health service | Community mental health services | Community. | Psychiatrist assessment. | Educational grant of the Consorzio Mario Negri Sud, an independent public–private research institute, which contributed the facilities for data collection and handling, as well as the services for data quality monitoring through its certified unit | Supported social activities.  Delivered by health professionals or natural volunteers.  Control: usual care | 3-6 months | 24 months. |
| Sheridan, 2015 | Ireland | Circulation of promotional materials and referral directly from mental health services. | Referral from mental health services and self-referral. | Community. | Structured data collection interviews - quantitative. | Health Research Board grant number, 2006/HRB/RPG06. The funder was not involved in the study design, data gathering, analysis or writing of the final report. | Supported social activities and friendship plus financial stipend. Facilitated by volunteer partner.  Control: financial stipend only to support social activity. | 9 months. Two hours per week. | 10 months. |
| Thorup, 2006 | Denmark | Referral by health professional | Health services. | Community | Structured interviews and assessments by independent, trained professionals. | The Danish Ministry of Health, the Danish Ministry of Social Affairs, the Danish Medical Research Council, Copenhagen Hospital Corporation, Aarhus County, the Wørzner Foundation and the University of Copenhagen have funded the OPUS-trial. | Assertive community treatment.  Delivered by health professionals.  Control: treatment as usual. | 12 months. Took place biweekly in the first 2 months and then once a week in the following 10 months | 24 months. |
| Castelein, 2008 | The Netherlands | Not stated. | Mental Health Care Centres. | Mental Health Care Centrse. | Independent questionnaire completion with a professional available to support. | This study was granted by Zon Mw (the Netherlands Organisation for Health Research and Development), the Rob Giel Research Center, and The Roos Foundation. | Closed peer support group.  Delivered by: Closed peer support group facilitated by a nurse.  Control: waiting list | 8 months. 16 sessions of 90 min biweekly over 8 months | 8 months. |
| Calsyn, 1998 | USA | Not stated. | Emergency shelters/health serivces/A&E. | Community. | Interviews by master’s level psychologists and social workers. | This project was supported by grants from the National Institute of Mental Health | Assertive community treatment.  Delivered by health professionals.  Control: treatment as usual (ACT vs drop in centre vs outpatient setting) | Unspecified. | 18 months. |
| Tempier et al 2012 | England | Not stated. | Early onset trial. | Community. | Questionnaires administered by trained researcher. | None stated. | Assertive community treatment.  Delivered by health professional.  Control: treatment as usual | 12 months. | 18 months. |
| Johnson et al 2018 | England | Referral by health professional | Eligible participants had been on crisis resolution team caseloads for at least a week, and had capacity to give informed consent | Community. | Questionnaires delivered by trained researchers. | Undertaken as part of the CORE Study, which was funded by the National Institute for Health Research under its Programme Grants for Applied Research programme (reference RP-PG-0109-10078). | One-to-one recovery focussed work to support community integration and social network enhancement by peer workers.  Delivered by peer workers.  Control: personal recovery workbook | Ten individual sessions of 1 hour each with a peer support worker. Sessions took place roughly once per week, aiming to conclude within 4 months. | 18 months. |
| Ammerman (2013) | USA | Referral by health professional | Referral by home visitor. | Home | Standardised outcome measures. | Supported by Grant R34MH073867 from the National Institute of Mental Health | One-to-one cognitive behavioural therapy. with a social network focus.  Delivered by health professionals  Control: Treatment as usual. | Treatment consisted of 15 sessions that were scheduled weekly and lasted 60 min plus a booster session one-month post-treatment. | 3 month follow-up. |
| Rivera, 2007 | USA | Recruitment in inpatient units by researchers. | Inpatient units. | Combination of clinic based service provision and that provided in the general community by peer workers. | Assessment of hospital records, monthly interviews using self-report instruments.  Research assistants who were blind to the treatment assignments collected all interview data, except for the social network measures, which were collected by the professional staff.. | This research was supported by funds furnished by the New York City Department of Health and Mental Hygiene, the New York State Office of Mental Health, and the New York City Health and Hospitals Corporation. This article has not been officially reviewed or cleared by any of the funding sources. | Supported social activity.  Delivered by volunteer peers  Control: Treatment as usual. |  | 12 months. |

| **Participants** | | | | | | |
| --- | --- | --- | --- | --- | --- | --- |
| **STUDY ID (Author last name, year)** | **N RECRUITED** | **AGE RANGE** | **MEAN AGE** | **ETHNICITY** | **GENDER  (N or %)** | **PRIMARY DIAGNOSIS** (or MH measure; note if self-report) |
| Terzian et al 2013 | 357 | 18-45 | Not Stated. | Not stated. | CONTROL: 72.1% Male/27.9% Female -  INTERVENTION: 65.3% Male/34.7% Female - | Schizophrenia spectrum |
| Sheridan, 2015 | 118 | Not stated. | 51 | Not stated. | 52.3% (n=56) - female | Serious mental illness as denoted by International Classification of Diseases 10 (ICD-10) criteria for schizophrenia, schizophrenia spectrum and delusional disorders (F20-29), mood (affective) disorders (F30-39), neurotic, stress-related and somatoform disorders (F40-40) and mental and behavioural disorders due to psychoactive substance use – drug-induced psychosis (F10-19; ICD-10). |
| Thorup, 2006 | 547 | 18-45 | Not stated. | Not stated. | 40.9% (n=224) female. | ICD-10 diagnoses of schizophrenia, acute psychoses, schizotypal disorder, schizoaffective disorder or other delusional disorders in the F20- spectrum |
| Castelein, 2008 | 106 | Not stated, | CONTROL: 39.4  INTERVENTION: 37.8 | Not stated.. | CONTROL: 63% MALE.INTERVENTION: 68% MALE | Schizophrenia or a related psychotic disorder. |
| Calsyn, 1998 | 165 | None | Study 1: 33.66; Study 2: 34.76 | Study 2: 45% Caucasian; 54.5% African-American | 58% male | **Study 2:** The sample had DSM-IIIR AXIS I diagnoses. |
| Tempier et al 2012 | 144 | 16-40 | 25.85 | White Specialised Care (SC) (n=24) 42% Standard care (ST) (n=12) 24%  Black SC (n=24) 42% ST (n=29) 58%  Other SC (n=9) 16% ST (n=9) 18% | SC: (n=30) 53% Male. ST (n=39) 78% Male. | First episode of psychosis. |
| Johnson et al 2018 | 441 | Not stated. | 40 | White: Intervention (65%) control (65%)  Black (UK, African, Caribbean, and Other) intervention (20%) control (19%)  Asian (UK, south Asian, Chinese, and Other) intervention (6%) control (6%)  Other intervention (9%) control (11%) | Male intervention 88/220 (40%) control 87/218 (40%) Female intervention 132/220 (60%) control 131/218 (60%) | All diagnoses. |
| Ammerman (2013) | 93 | 16–37 years. | 21.9 | White: IH-CBT: 30 (63.8%); SHV: 28(60.8%)  African American: IH-CBT: 14(29.9%); SHV: 16(34.8%) Native American: IH-CBT: 1(2.1%); SHV: 0 (0.0%) Native Hawaiian or other Pacific Islander: IH-CBT 1(2.1%); SHV: 1(2.2%) Bi-racial: IH-CBT: 1(2.1%); SHV: 1(2.2%) Latina: IH-CBT: 3(6.4%); SHV: 4(8.7%)  None: IH-CBT: 44(93.6%); SHV: 42(91.3%) | 100% Female. | Major Depressive Disorder (MDD) |
| Rivera, 2007 | 255 | Not reported. | 38.3 years | **Race or ethnicity** Caucasian: 58 (29%) African American 35 (17%) Hispanic 62 (31%) Other: 48 (24%) | Female: 100 (49%) | All participants had a diagnosis of a psychotic or mood disorder on axis I. |

| **Outcomes** | | | | | |
| --- | --- | --- | --- | --- | --- |
| **STUDY ID:** | **Social network measure** | **Social network measure data** | **Other relevant social network data.** | **Other outcomes** | **Authors conclusion** |
| Terzian et al 2013 | Social network size: Number, frequency, importance, or closeness of relationships | A social network improvement was observed at year 1 in 25% of the patients allocated to routine treatment and in 39.9% of those allocated to the experimental arm (OR 2.0, 95% CI 1.3 to 3.1; adjusted OR 2.4, 95% CI 1.4 to 3.9).  The difference remained statistically significant at year 2. | Participants attributed higher value to arm’s length relationships rather than friendships or confiding relationships.  Results suggest that improving social networking produces beneficial effects in patients with a better clinical prognosis. E.g. a good clinical prognosis might anticipate a good response in terms of social network improvement. This is not the case for the other outcomes, as the experimental treatment appears to be effective by and large regardless of improvements or worsening of work or activities of daily living. | No significant differences emerged for any of the other end points (Brief Psychiatric Rating Scale/Global Assessment of Functioning/Self-care/Activities of daily living/Hospitalisation/Work). However, patients with 1 or more other areas of improvement at year 1 and 2 showed a statistically significant social network improvement. | The activation of social networks as an activity integrated with standard psychiatric care is practicable, without added economic and organizational costs, and appears to produce an effect persisting well beyond its implementation. |
| Sheridan, 2015 | Practitioner Assessment of Network Type | At baseline, approximately 54% of the partnered and 57% of the un-partnered group were living in social networks with relatively limited contact with friends or neighbours and approximately 40% of both groups in the two most socially vulnerable networks. The main change over the course of the study was the extent to which participants reported having contact with friends on a weekly basis. However, the proportion of respondents in both groups who had no friends remained reltively unchanged.  Although there was variability in the extent to which the social networks of the control and intervention groups changed through- out intervention, there was a decline in the proportion of participants in the two most vulnerable networks. |  | **Beck depression Inventory:** No significant difference between groups..  **Loneliness:** Social and family loneliness decreased significantly over time for both groups. Romantic loneliness scores also decreased, the decrease was not statistically significant.  **Rosenberg’s self-esteem measure:** Both groups demonstrated good levels of self-esteem and levels remained consistent for both groups throughout the study.  **Social functioning:** involvement in social recreational activities increased significantly over time for both groups and there was a slightly higher level of recreational involvement in the partnered group; however, the difference between both groups was not statistically significant. | The intervention showed no statistical differences between the control and intervention groups on primary or secondary outcome measures. The stipend and the stipend plus volunteer partner led to an increase in recreational social functioning; a decrease in levels of social loneliness, in depression and in the proportion living within a vulnerable social network. |
| Thorup, 2006 | Social network size: Number of contacts with family and friends | The type of treatment did not affect the social network size after 2 years of intervention, since the distribution was not significantly different for ST and IT. At 2-year follow-up, IT patients had on average 4.3 contacts with family members in the previous month, while ST patients had on average 4.7 contacts (P = 0.28). The average number of contacts with friends during previous month was 3.8 for both IT and ST patients. | The final models included both age and network size at entry, but while the final model for family-network size included male gender and disorganised dimension, the friends-network size model included negative symptoms and A-level status as the significant variables. The univariate models show that dependence syndrome leads to less family contact, while not having completed high school and poor academic premorbid functioning do not. The geo- graphical variable ‘site’ only has significant impact on number of friends. | None reported. | Premorbid function- ing, network size at entry and DUP is closely related to small social network size. The integrated psycho- social treatment programme was not sufficient to address this problem. |
| Castelein, 2008 | Self-developed list [Personal Network Questionnaire (PNQ)] asking for information on the frequency of contacts with named family, friends, and members of the peer support group. | Participants had a significant increase in contact with peers outside of the sessions (P = 0.03) and on esteem support (i.e. asked for advice, received a compliment, asked for help; P = 0.02) in comparison with the WLC condition (56% improvement vs 31% improvement). The positive effect on peer contact did not generalize to other relationships; for instance, contact with family and friends. | More negative symptoms at baseline (P = 0.02) and more distress caused by these symptoms (P = 0.05) predicted improved psychological health, but not on social relations (P = 0.01). More distress caused by positive symptoms (P = 0.05) and a longer duration of illness (P = 0.06) predicted improved social relations. Those with higher distress from negative symptoms had significantly less chance of improving on social relations (P = 0.01). | **Symptomology:** the participants in the experimental condition had statistically significant, fewer negative symptoms (P = 0.02) and less distress from these symptoms (P = 0.04) in comparison with the participants in the control condition. In addition, no between-condition differences were found in hospitalization rates (P = 0.28) during the intervention.  For self-efficacy, self- esteem, and self-reported quality of life, no between-condition differences were found, but participants in both conditions improved over the study period.  The high attender group significantly improved on social support, self-efficacy, and quality of life compared with the low attender group. | This first RCT on peer support groups for people with psychosis demonstrates that this intervention is effective in improving their social network by encouraging mutual relationships and in enhancing their appraisal support. |
| Calsyn, 1998 | Social network size; Arizona Social Support Interview Schedule with additional items; Personality and Social Network Adjustment Scale | **Study 2:** There was a significant treatment group effect on the network size variables, Wilks Lamda (6, 252) 4 .84, p , .0001. Although there was no significant difference between treatment groups in terms of the size of the natural support network, there was a significant difference in the size of the professional network, F (2, 126) 4 10.47, p , .0001. | **Study 2:** There were no significant treatment group differences on the emotional, ad- vice, recreational and conflict dimensions. However, there was a significant treatment group difference on the material assistance dimension, F (2, 122) 4 7.20, p , .001.  There were no significant effects of treatment group, time, or treatment group by time on the qualitative measures of social relation- ships. There was a significant effect of treatment on the interviewers’ ratings of the adequacy of the social network, Wilks Lambda (6, 232) 4 .73, p , .0001. | None reported. | Only limited support for the hypotheses that ACT increases the social support of people with severe mental illness who are homeless. The most consistent finding across both studies was that clients served by ACT programs had significantly larger professional support networks than clients served by the other programs. This finding is consistent with the ACT treatment approach that calls for intensive staff intervention and a team approach. |
| Tempier et al 2012 | Social network size; functional adequacy of social support as measured by the gap between ideal and perceived levels of support. | The mean network size was not statistically different between groups at 6 months, although there was a trend toward bigger networks among specialized care patients. When members of the patient’s network were classified by role, for example, parent or sibling, participants in the specialized care group tended to report more responses for each role, suggesting that their net- works were denser.  At 18 months, the intervention group had a significantly larger network than the standard care group. | A comparison of emotional and practical support subscales of the SOS showed no difference between groups in ideal and perceived levels of support. For all subscales, the perceived level of support was less than the ideal level. | No significant differences were found in either clinical outcome measures between the two treatment groups at six- month follow-up.  At 18 month follow-up the intervention group scored significantly better on the negative PANSS, total PANSS, and GAF. | Early intervention by using an ACT model of care may improve clinical results by reestablishing or maintaining bonds between patients and family, friends, and acquaintances. |
| Johnson et al 2018 | Lubben Social Network Scale | At 4 and 18 months, there was little  evidence of any effect; the difference in social networks favoured the intervention but it was not statistically significant | None. | Readmission to acute care within 1 year was significantly lower in the intervention group than in the control group. Time to readmission was significantly longer in the intervention than in the control. However, the number of days in acute care was not significantly different. Participants in the intervention group had fewer days in acute care than did participants in the control group, but the difference was not significant.  At 4 months of follow-up, overall satisfaction with mental health-care received was greater in the intervention group than in the control group.  There was also a significant difference in self-rated recovery favouring the intervention, but the difference was not significant in sensitivity analysis with adjustment for predictors of missingness. | Our findings suggest that peer-delivered self-management reduces readmission to acute care, although admission rates were lower than anticipated and confidence intervals were relatively wide. The complexity of the study intervention limits interpretability, but assessment is warranted of whether implementing this intervention in routine settings reduces acute care readmission. |
| Ammerman (2013) | Social Network Index. Network Size Subscale; Network Diversity Subscale; Network Embeddedness Subscale. | No group differences were found in size of and involvement with social networks. For each scale, both groups either remained stable over time or showed increases over time. | Those receiving IH-CBT reported increased social support over time relative to those in the SHV condition. Effect sizes were modest at post-treatment (ES=0.38) but increased at follow-up (ES=0.65).  Improvements were seen in affiliative and belonginess aspects of social support, in contrast to tangible support which was statistically non-significant. Findings were not moderated by clinical features of depression or home visiting parameters. | Subjects receiving IH-CBT reported decreased psychological distress at post- treatment (ES=0.77) and follow-up (ES=0.73). Examination of types of psychological distress indicated broad improvements at both time points. | IH-CBT is effective in reducing psychological distress and improving perceived social support in depressed mothers receiving home visiting. IH-CBT is a feasible, readily adopted treatment that is compatible with multiple home visiting models. As a result it is a promis- ing approach to help depressed mothers in home visiting. Additional interventions may be needed to support depressed mothers in building sizable and stable social networks. |
| Rivera, 2007 | Pattison Network Inventory (31,32). This interview assessed social network size, total number of social contacts, degree of reciprocity of relationships, density of the social network, and the number of times the client was helped or had helped others in his or her network. | Clients receiving peer-assisted care showed a significant increase in the number of contacts from baseline to 12 months. Follow-up analyses revealed that this effect was due to increased contact with peer assistants and professional staff, not with family and outside friends. There were also significant improvements for all conditions in several other network measures as indicated by reliable main effects of time: total number of others involved in social activities, total number of others who helped client, total number of others helped by client, and network density.  Peer-assisted care showed the greatest increase in self-reported social contacts with consumer and professional staff. Peer assistants provided planned activities and regularly scheduled home visits to enhance the social network. These increases did not extend to kin social contacts, |  | Data indicate that although the three programs had distinct patterns of services, they yielded the same general pattern of improvement over time on a variety of measures: symptoms, health care satisfaction, and various ratings of the quality of life. Clients in the three programs also showed similar but small changes in measures of social network behavior. No one program emerged as categorically superior to the others. | Although the work of peers enhanced the social networks of consumers, this did not translate into measurable changes in treatment outcome. |

| **Risk of bias assessments** | | | | | |
| --- | --- | --- | --- | --- | --- |
| **STUDY ID:** | **Selection bias** | **Allocation bias: comparability at baseline and concealment** | **Blinding of outcome assessment** | **Incomplete outcome assessment** | **Adherence to intervention** |
| Terzian et al 2013 | Low | Low | High | Low | Unclear |
| Sheridan, 2015 | Low | Low | Low | High | Unclear |
| Thorup, 2006 | Unclear | Low | Unclear | High | Unclear |
| Castelein, 2008 | Low | Low | High | Low | Low |
| Calsyn, 1998 | Unclear | Unclear | Unclear | Unclear | Unclear |
| Tempier et al 2012 | Low | Low | Low | Unclear | Unclear |
| Johnson et al 2018 | High | Low | Low | Low | Low |
| Ammerman (2013) | Low | Low | Unclear | Low | Unclear |
| Rivera, 2007 | Unclear | Low | Unclear | Low | Unclear |

High risk: One high-risk classification within domains

Low risk: All low-risk classifications across domains.

Unclear: One unclear classification within domains.
